# Ultrasonographic and Shear Wave Elastographic Assessment of the Mentalis Muscle: Associations with Mandibular Symphysis and Craniofacial Morphology

**DOI:** 10.3390/diagnostics16213527

**Published:** 2026-10-27

**Authors:** Merve Berika Kadıoğlu, Meyra Durmaz, Nisa Yıldız, Gözde Haksayar, Mehmet Alp Eriş, Cengiz Evli, Mahmut Kadıoğlu

**Affiliations:** 1 Department of Orthodontics, Faculty of Dentistry, Ankara University, 06560 Ankara, Türkiye; mkadioglu@ankara.edu.tr (M.B.K.); dtmeyradurmaz@gmail.com (M.D.); 2 Department of Orthodontics, Faculty of Dentistry, Nevşehir Hacı Bektaş Veli University, 50300 Nevşehir, Türkiye; nlnsay2757@gmail.com; 3 Dentomaxillofacial Radiology, Institute of Health Sciences, Ankara University, 06560 Ankara, Türkiye; gozde.haksayar@hotmail.com; 4 Dentomaxillofacial Radiology, Faculty of Dentistry, Ankara University, 06560 Ankara, Türkiye; cevli@ankara.edu.tr; 5 Department of Oral and Maxillofacial Surgery, Faculty of Dentistry, Ankara University, 06560 Ankara, Türkiye; 6 Independent Researcher, 06560 Ankara, Türkiye; drmahmutkadioglu@gmail.com

**Keywords:** cephalometry, craniofacial morphology, mandibular symphysis, mentalis muscle, shear wave elastography, ultrasonography

## Abstract

**Background/Objectives**: This study aimed to evaluate the ultrasonographic thickness and shear wave elastography (SWE)-derived stiffness of the mentalis muscle across different sagittal skeletal patterns and to investigate their relationships with mandibular symphysis morphology and cephalometric variables. **Methods**: Forty-eight adults aged 18–35 years were classified according to ANB angle into Class I, Class II, and Class III groups, with 16 participants in each group. Mentalis muscle thickness and stiffness were assessed bilaterally at lateral, intermediate, and medial regions using ultrasonography and SWE. Cephalometric skeletal, dental, soft-tissue, and symphyseal measurements were evaluated. Group comparisons were performed using one-way ANOVA or Welch’s ANOVA, as appropriate, and within-group associations were assessed using Pearson correlation analysis with Benjamini–Hochberg false discovery rate correction applied separately to elastography and muscle-thickness correlation families. **Results**: Mentalis muscle thickness did not differ significantly among the skeletal groups. Right intermediate elastography was the only ultrasonographic parameter showing a significant overall intergroup difference (*p* = 0.011, η^2^ = 0.182), with Bonferroni-adjusted pairwise comparisons showing significantly higher values in Class II than in Class I (adjusted *p* = 0.012). After FDR correction, selected associations between elastographic stiffness and skeletal, dental, or soft-tissue variables remained significant, whereas no muscle-thickness correlations remained significant. These associations varied across skeletal groups and were considered exploratory. **Conclusions**: Resting ultrasonographic characteristics of the mentalis muscle showed no consistent association with mandibular symphysis morphology across sagittal skeletal patterns. Mentalis muscle thickness was comparable among the groups, whereas elastographic stiffness demonstrated only a limited and region-specific between-group difference. Selected associations with skeletal and soft-tissue variables were observed; however, these exploratory findings should not be interpreted as evidence of skeletal-class-specific biological mechanisms. Larger studies incorporating functional assessments of the perioral musculature are needed to confirm these findings.

## 1. Introduction

Morphological evaluation of the mandibular symphysis is important in orthodontic diagnosis and treatment planning, particularly for determining dentoalveolar boundaries, mandibular incisor positioning, and lower facial esthetics. The symphysis is a dynamic anatomical unit associated with sagittal jaw relationships, vertical growth pattern, and mandibular incisor inclination [1,2]. Its morphology is influenced by genetic, adaptive, and neuromuscular factors, as well as incisor inclination and overbite [3]. Symphyseal dimensions vary among sagittal skeletal relationships, with Class III individuals showing distinct patterns of dentoalveolar compensation and differences in symphyseal morphology and height [2,4].

In addition to hard tissue characteristics, soft tissues and muscular function may contribute to dentoalveolar adaptation and mandibular symphysis morphology. The perioral musculature contributes to the functional equilibrium surrounding the dentition, and recent evidence suggests that differences in oral muscle pressure and muscle balance are associated with malocclusion and dentoalveolar relationships [5]. Masticatory muscle morphology and function are also associated with craniofacial skeletal patterns, with differences in muscle thickness, activity, and stiffness reported among facial types [6,7]. Long-face individuals generally exhibit thinner muscles and lower functional capacity, whereas short-face individuals present with more robust muscular morphology [8].

The mentalis muscle plays an important role in lower lip function and lip sealing. Increased mentalis activity has been reported in individuals with lip incompetence and skeletal discrepancies, and recent electromyographic evidence also suggests associations between mentalis activity, vertical craniofacial morphology, and incisor position [9,10,11]. Finite element analysis has further suggested that excessive mentalis activity may alter stress distribution in the anterior mandibular dentoalveolar region and influence local morphology [12]. Thus, the mentalis muscle may contribute to both the functional environment and biomechanical characteristics of the anterior mandibular region.

However, evidence directly linking the mentalis muscle to mandibular symphysis morphology remains limited. Pintavirooj et al. found that mentalis hyperactivity was associated with increased symphyseal height and reduced alveolar width [13]. Most available studies rely on electromyography, while structural assessments of the mentalis muscle across sagittal malocclusion patterns remain scarce.

Although electromyography (EMG) is widely used to evaluate muscle function, its reliability may be affected by electrode placement, signal cross-talk, and measurement standardization [14,15]. Ultrasonography provides a non-invasive and reproducible assessment of muscle thickness and structure [16,17], while shear wave elastography (SWE) enables quantitative evaluation of muscle stiffness and mechanical properties [18]. Together, these methods allow a comprehensive assessment of muscle morphology and biomechanics.

However, few studies have investigated the relationship between the ultrasonographic and elastographic characteristics of the mentalis muscle and mandibular symphysis morphology. Most previous research has focused on the masseter and temporalis muscles, leaving the mentalis muscle largely unexplored across different sagittal malocclusion patterns.

Therefore, the aim of the present study was to evaluate the relationship between ultrasonographic thickness and elastography-derived mechanical properties of the mentalis muscle and mandibular symphysis morphology, together with lateral cephalometric measurements, in individuals with different sagittal malocclusion patterns.

The null hypothesis of this study was that the ultrasonographic thickness and elastographic characteristics of the mentalis muscle would not be associated with mandibular symphysis morphology or other cephalometric variables and would not differ among sagittal malocclusion groups.

## 2. Materials and Methods

### 2.1. Study Design and Ethical Approval

This observational cross-sectional study was conducted at the Department of Orthodontics, Faculty of Dentistry, Ankara University, Ankara, Türkiye. The study protocol was approved by the Institutional Ethics Committee of Ankara University (Approval Date: 3 July 2023; Approval No. 36290600/50/2023). Written informed consent was obtained from all participants before enrollment.

### 2.2. Participants and Sample Size

Sample size was calculated using G*Power version 3.1 (Heinrich Heine University, Düsseldorf, Germany). Assuming a one-way analysis of variance (ANOVA), an effect size (f) of 0.60, a significance level of 0.05, and a statistical power of 95%, the minimum required sample size was calculated as 48 participants. Accordingly, 48 individuals were included, with 16 participants allocated to each group.

A total of 48 adults aged between 18 and 35 years were classified into three sagittal skeletal malocclusion groups according to the ANB angle: Class I (0–4°), Class II (>4°), and Class III (<0°), with 16 participants in each group. The ANB angle was used as the predefined criterion for sagittal skeletal classification, while Wits appraisal was recorded as a complementary measure of sagittal jaw relationship but was not used to redefine the study groups.

### 2.3. Eligibility Criteria

Participants were included if they fulfilled all of the following criteria:

- Age between 18 and 35 years;
- Fully erupted permanent dentition (excluding third molars);
- No previous orthodontic treatment;
- Availability of pretreatment lateral cephalometric radiographs.

Participants were excluded if any of the following conditions were present:

- Congenital tooth agenesis or supernumerary teeth (excluding third molars);
- Severe dental wear;
- Craniofacial anomalies;
- History of trauma, tumor, or infection affecting the maxillofacial region;
- Systemic diseases or syndromes affecting craniofacial development.

### 2.4. Cephalometric Analysis

**Figure 1:**
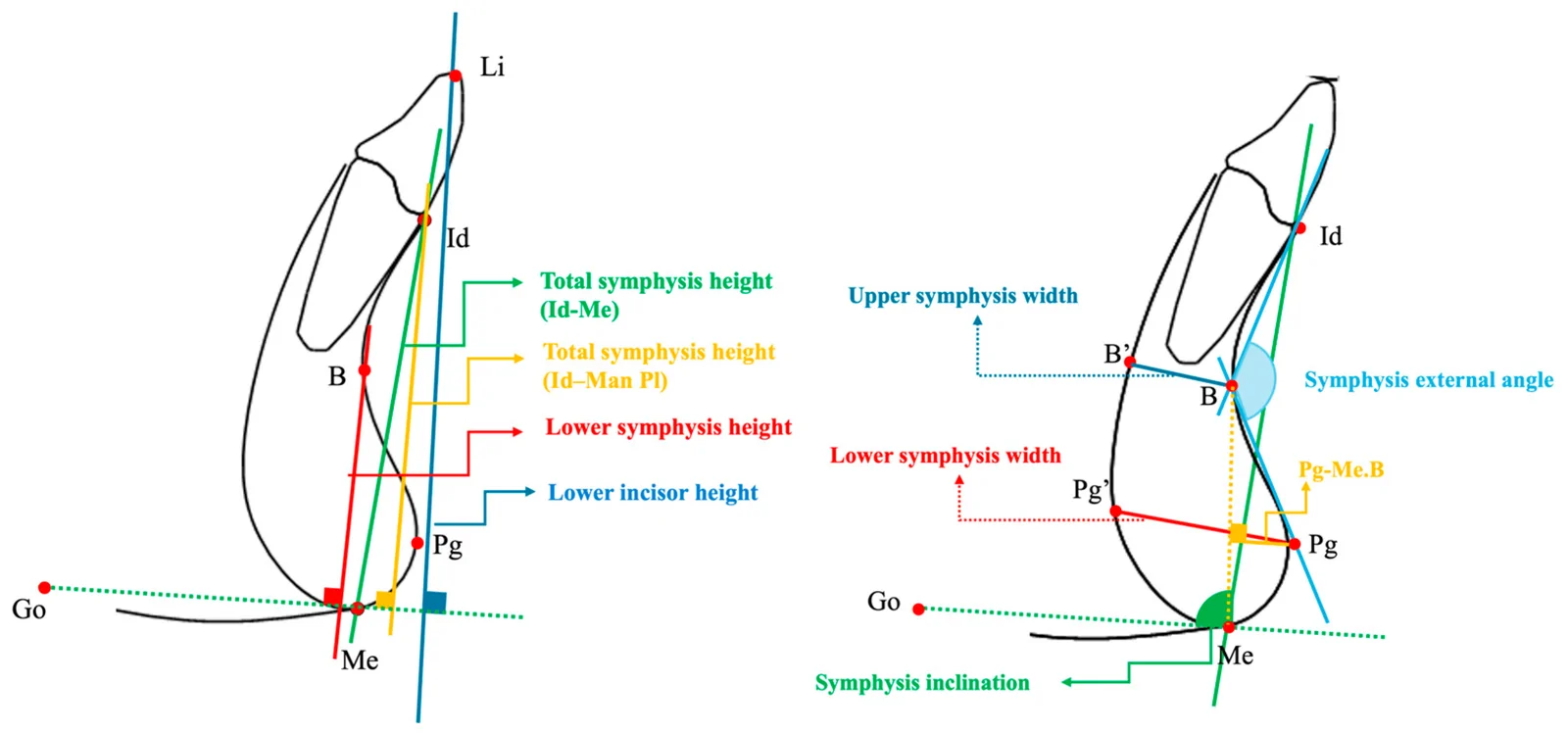
Cephalometric measurements used for the evaluation of mandibular symphysis morphology. Id, infradentale; Me, menton; B, point B; Pg, pogonion; Go, gonion; Li, lower incisor; Man Pl, mandibular plane. B′ and Pg′ represent the corresponding points on the inner cortical border of the symphysis opposite B and Pg, respectively. Linear measurements included total symphysis height (Id–Me and Id–Man Pl), lower symphysis height (B–Man Pl), lower incisor height (Li–Man Pl), anterior symphyseal prominence (Pg–NB and Pg–Me.B), and upper and lower symphysis widths (B–B′ and Pg–Pg′, respectively). Pg–Me.B represents the perpendicular distance from Pg to the line connecting B and Me. Angular measurements included the symphysis external angle (Id–B–Pg), reflecting symphyseal concavity, and symphysis inclination (Id–Me/Man Pl), reflecting the inclination of the symphysis relative to the mandibular plane.

Standardized pretreatment lateral cephalometric radiographs were analyzed digitally using Dolphin Imaging software version 11.8 (Dolphin Imaging & Management Solutions, Chatsworth, CA, USA). Skeletal, dental, soft tissue, and mandibular symphysis measurements were performed according to previously validated cephalometric landmarks and definitions described in the literature. Skeletal variables included sagittal and vertical skeletal relationships, dental variables comprised incisor position and inclination together with overjet and overbite, and soft tissue variables included lip position and the nasolabial angle. Mandibular symphysis morphology was evaluated using linear and angular measurements characterizing symphyseal height, width, anterior prominence, concavity, and inclination relative to the mandibular plane. The principal symphyseal measurements are illustrated in Figure 1, with additional definitions provided in the figure legend.

The conventional cephalometric measurements evaluated in the study are presented in Supplementary Figure S1.

### 2.5. Ultrasonographic Evaluation

The mentalis muscle was evaluated using a high-resolution ultrasound system (ACUSON S2000, Siemens, Munich, Germany) equipped with a 9L4 linear-array transducer (4–9 MHz), which was used for both B-mode thickness measurements and shear-wave elastography. Imaging settings were consistent with those previously described for the masseter muscle using the same ultrasound system (focal range, 0.5–2.5 cm; image depth, 5.5 cm; dynamic range, 65–90 dB) [19]. All ultrasonographic examinations were performed by the same dentomaxillofacial radiologist with four years of experience, who did not perform the intraoral examination; skeletal classification was determined separately from the lateral cephalometric radiographs. Lateral cephalometric radiographs and ultrasonographic examinations were obtained on the same day for all participants.

Participants were examined in the supine position with the head maintained in a relaxed natural posture. Immediately before acquisition, participants were asked to contract and release the lips twice; this brief activation also facilitated identification of the mentalis muscle on the real-time ultrasound image, as lip pressing has previously been used for this purpose in facial muscle ultrasonography [16]. Participants then maintained their lips lightly closed in a relaxed rest position without voluntary lip or chin movement, and measurements were obtained immediately thereafter with the muscle at rest. All participants were able to maintain passive lip closure without strain; therefore, no interlabial gap was present, and interlabial distance was not measured. Muscle relaxation was additionally confirmed by palpation of the chin before imaging.

A substantial amount of water-based ultrasound gel was applied between the skin and the transducer to avoid compression of the superficial soft tissues, and the transducer was held with very light, feather-like skin contact without active pressure [16,19,20]. A standoff pad was not used, because aqueous standoff pads have been reported not to improve the visualization of facial muscles [16]. To maintain stable skin contact, the examiner rested the heel of the hand or the little finger on the participant’s head and avoided resting the arm on the participant’s chest to minimize interference from breathing movements [19].

The transducer was positioned transversely over the mentalis region, centered on the soft-tissue pogonion, and held perpendicular to the skin surface, as previously described for ultrasonographic assessment of the mentalis muscle [16,20]. The mental protuberance, mandible, and contralateral muscle were used as anatomical landmarks for muscle identification [16]. The transducer angle was then adjusted until the anterior cortex of the symphysis appeared as a sharp, continuous hyperechoic line, an approach previously used for the masseter muscle [19], and this orientation was maintained throughout the examination. Because a single transverse image displayed the entire muscle bilaterally over the convex symphysis, all regions were measured from the same transducer position, which limited angle variation related to the curvature of the chin.

To characterize the transverse morphology of the mentalis muscle rather than relying on a single focal measurement, bilateral assessments were performed at three predefined regions on each side: lateral, intermediate, and medial. On the B-mode image, each hemi-muscle extending from the midline at the pogonion to its lateral border was divided into three equal segments (medial, intermediate, and lateral from the midline outward). Measurements were obtained at the center of each segment, resulting in six regions per participant: right lateral, right intermediate, right medial, left medial, left intermediate, and left lateral. At each site, muscle thickness and shear-wave elastography measurements were obtained. Muscle thickness was recorded in centimeters (cm), whereas tissue stiffness was quantified in kilopascals (kPa).

**Figure 2:**
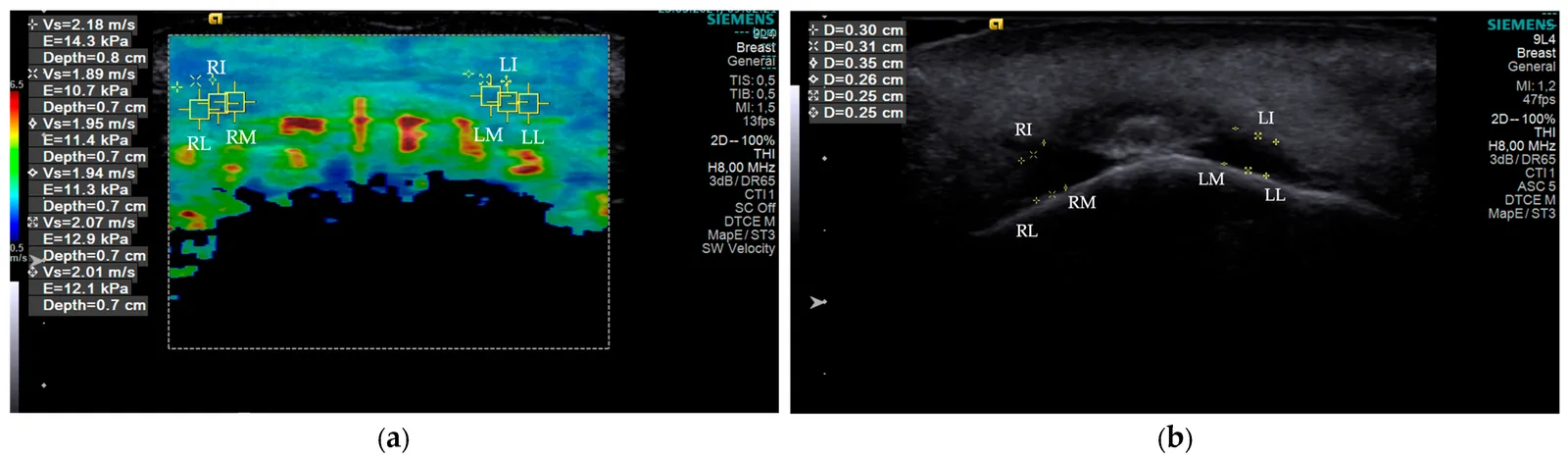
Representative ultrasonographic images of the mentalis muscle: (**a**) shear-wave elastography showing the predefined bilateral measurement regions; (**b**) B-mode ultrasonography showing muscle thickness measurements. Measurement regions are indicated as RL, right lateral; RI, right intermediate; RM, right medial; LM, left medial; LI, left intermediate; and LL, left lateral.

For elastographic assessment, the measurement region was positioned at the mid-thickness of the visualized mentalis muscle in each segment while avoiding inclusion of the overlying skin and underlying mandibular cortex. A fixed, system-defined ROI size was used throughout the examinations and was not altered between participants or measurement sites. The ROI was consistently positioned within the central portion of each predefined segment, and the same acquisition settings and ROI placement approach were used for all participants and measurement sites. Identical system settings were used for all participants and regions, and the depth of each measurement region from the skin surface was recorded automatically by the system. Measurements were obtained only when the muscle boundaries could be clearly identified on the ultrasound image. Each thickness and elastography measurement was performed twice at each site, and the mean of the two measurements was used for statistical analysis. The ultrasonographic acquisition protocol and representative thickness and elastography measurements are illustrated in Figure 2.

### 2.6. Reliability Assessment

To assess intra-examiner (inter-session) reliability, ultrasonographic measurements, including both muscle thickness and SWE-derived stiffness, as well as cephalometric measurements, were repeated in 15 randomly selected participants two weeks after the initial evaluation. Intra-class correlation coefficients (ICC) were calculated to determine measurement reliability. ICC values ranged from 0.89 to 0.95, indicating excellent intra-examiner reliability. Inter-examiner reliability was not assessed because all measurements were performed by the same examiner.

### 2.7. Statistical Analysis

Statistical analyses were performed using IBM SPSS Statistics version 26.0 (IBM Corp., Armonk, NY, USA). The normality of continuous variables was assessed using the Shapiro–Wilk test together with skewness and kurtosis values. Continuous variables are presented as mean ± standard deviation (SD) and 95% confidence intervals (CIs).

Differences among the three skeletal malocclusion groups were evaluated using one-way analysis of variance (ANOVA). Homogeneity of variances was assessed using Levene’s test. When the homogeneity assumption was satisfied and the overall ANOVA was statistically significant, pairwise comparisons were performed using Bonferroni-adjusted post hoc tests. For variables violating the homogeneity of variance assumption, Welch’s ANOVA followed by Games–Howell post hoc comparisons was used. Effect sizes for between-group comparisons were expressed as eta-squared (η^2^).

Pearson correlation analysis was used to evaluate the relationships between ultrasonographic mentalis muscle measurements and cephalometric variables within each skeletal malocclusion group. These within-group correlation analyses were considered secondary and exploratory. To account for multiple testing, *p* values were adjusted using the Benjamini–Hochberg false discovery rate (FDR) procedure, applied separately within each skeletal group for elastography and muscle thickness correlation families. Only correlations remaining significant after FDR correction were considered statistically significant. The 95% CIs for Pearson correlation coefficients were calculated using Fisher’s r-to-z transformation. Spearman rank correlations were additionally performed as a sensitivity analysis for associations that remained significant after FDR correction.

To assess the potential influence of vertical skeletal pattern on the intergroup difference in right intermediate elastography, separate general linear models were constructed using SN–GoGn and Björk sum as covariates. Group-by-covariate interaction terms were included to assess the homogeneity of regression slopes. Because significant interactions indicated violation of this assumption, conventional ANCOVA estimates assuming a common regression slope were not interpreted.

For correlation analyses, tests were two-tailed. A *p* value < 0.05 was considered statistically significant for group comparisons, whereas an FDR-adjusted *p* value (q) < 0.05 was considered statistically significant for the correlation analyses.

## 3. Results

A total of 48 participants were included in the study, with 16 individuals in each skeletal malocclusion group. The mean age was 23.19 ± 3.78 years in Class I, 24.81 ± 3.75 years in Class II, and 24.38 ± 3.44 years in Class III, with no significant difference among the groups (*p* = 0.436). The sex distribution was also comparable among the groups, with 10 females and 6 males in Class I, 8 females and 8 males in Class II, and 9 females and 7 males in Class III (*p* = 0.776) (Table 1).

Significant intergroup differences were observed for several sagittal and dentoalveolar measurements (Table 2). As expected, SNB, ANB, and Wits appraisal differed significantly among the three skeletal groups (all *p* < 0.001). Class III showed a greater gonial angle and lower IMPA and L1–NB measurements than Classes I and II. Significant differences were also observed for U1–NA measurements, overbite, and overjet. In contrast, SNA, SN–GoGn, Björk sum, and interincisal angle did not differ significantly among the groups. Detailed pairwise comparisons are presented in Table 2.

Significant intergroup differences were observed for lower lip position relative to the E-plane, upper lip position relative to the E-plane, and nasolabial angle (Table 3). Lower lip to E-plane values were significantly greater in Class II than in Class III (*p* = 0.010). Upper lip to E-plane values differed across all three groups, with Class II showing the highest values, followed by Class I and Class III (*p* < 0.001). The nasolabial angle was significantly greater in Class II than in Class III (*p* = 0.023). Lower and upper lip heights did not differ significantly among the groups.

Most linear symphyseal measurements did not differ significantly among the skeletal groups (Table 4). Significant intergroup differences were observed only for the symphysis external angle and symphysis inclination. The symphysis external angle was significantly greater in Class III than in Class II (*p* = 0.007), whereas no significant difference was observed between Class I and the other groups. Symphysis inclination was significantly lower in Class III than in both Class I and Class II (*p* < 0.001).

Right intermediate elastography was the only ultrasonographic parameter showing a significant overall difference among the skeletal groups (*p* = 0.011, η^2^ = 0.182). Bonferroni-adjusted pairwise comparisons showed significantly higher values in Class II than in Class I (adjusted *p* = 0.012), whereas the Class I–Class III (adjusted *p* = 0.083) and Class II–Class III (adjusted *p* = 1.000) comparisons were not statistically significant. No significant intergroup differences were observed for the remaining elastography measurements or for any mentalis muscle thickness measurements (all *p* > 0.05) (Table 5).

After FDR correction, two significant correlations remained in the Class I group and three in the Class II group. In Class I, L1–NB distance was negatively correlated with right lateral elastography (r = −0.828, q = 0.013), while upper lip height was also negatively correlated with right lateral elastography (r = −0.766, q = 0.049). In Class II, ANB was positively correlated with left intermediate (r = 0.846, q = 0.007) and left medial elastography (r = 0.766, q = 0.032), while SNA was positively correlated with right intermediate elastography (r = 0.768, q = 0.032). In Class III, FDR-significant associations included positive correlations of SN–GoGn and Björk sum with several elastography measurements and negative correlations of lower lip position relative to the E-plane with right lateral and right medial elastography. No muscle thickness correlations remained significant after FDR correction. The full correlation matrices for the Class I, Class II, and Class III groups are provided in Supplementary Tables S1–S3, respectively, while Table 6 summarizes only those associations that remained statistically significant after FDR correction. For the 12 associations that remained significant after FDR correction, Spearman sensitivity analyses showed the same direction of association, and all remained statistically significant (Table 6).

Additional general linear models were fitted to explore whether the between-group difference in right intermediate elastography varied according to vertical skeletal morphology. Significant group × covariate interactions were observed in the models including SN–GoGn (F(2, 42) = 4.713, *p* = 0.014, partial η^2^ = 0.183) and Björk sum (F(2, 42) = 3.359, *p* = 0.044, partial η^2^ = 0.138). Thus, the homogeneity-of-regression-slopes assumption was not satisfied, and conventional ANCOVA estimates assuming a common slope were not interpreted. The group × covariate interaction results are provided in Supplementary Table S4.

## 4. Discussion

This study evaluated the ultrasonographic thickness and elastographic characteristics of the mentalis muscle across different sagittal skeletal patterns and investigated their relationships with mandibular symphysis morphology and cephalometric variables. The findings suggest that the association between the ultrasonographic characteristics of the mentalis muscle and craniofacial morphology is neither direct nor unidirectional; rather, selected exploratory associations were observed between elastographic properties and skeletal or soft-tissue variables.

The cephalometric findings related to symphysis morphology were generally consistent with the existing literature. Symphysis inclination was lower in the Class III group, whereas the external symphysis angle was lower in the Class II group; however, linear symphyseal dimensions did not differ significantly among the sagittal skeletal groups. Differences in symphysis inclination and anterior contour accompanying sagittal skeletal patterns have been described as part of dentoalveolar compensation mechanisms. In Class III malocclusion, this compensation is frequently accompanied by lingual inclination of the mandibular incisors [1,2,21]. The lower IMPA and L1-NB values observed in the Class III group are also consistent with this compensatory pattern.

Nevertheless, these dentoalveolar and symphyseal characteristics were not clearly reflected in the resting ultrasonographic features of the mentalis muscle. This finding differs from that of Pintavirooj et al. [13], who reported an association between mentalis hyperactivity, increased symphyseal height, and reduced alveolar width. However, the two studies did not assess the same physiological condition. Mentalis hyperactivity represents a functional state that becomes particularly evident during lip closure, whereas the present study evaluated the muscle at rest in participants who were not selected according to lip competence. Moreover, symphyseal morphology in adulthood reflects the cumulative long-term effects of factors such as growth pattern, incisor position, and vertical dental relationships [1,3]. Therefore, the present resting measurements cannot be used to directly evaluate the functional role of the mentalis muscle in relation to symphyseal morphology. In particular, the absence of group differences in muscle thickness and most elastography measurements should not be interpreted as evidence against a functional contribution of the mentalis muscle, because muscle activity, lip-seal behavior, and lip competence during function were not assessed.

Mentalis muscle thickness did not differ significantly among the sagittal skeletal groups. The mentalis is a relatively thin muscle that exhibits considerable interindividual morphological variation. Ultrasonographic and anatomical studies have described fused dome-shaped, separated dome-shaped, and flat configurations [22,23]. This marked anatomical variability, together with the limited thickness of the muscle, may make subtle differences among sagittal skeletal groups more difficult to detect.

Evidence regarding the relationship between the ultrasonographic characteristics of the mentalis muscle and craniofacial skeletal pattern remains limited, with existing studies focusing primarily on the anatomical morphology and local characteristics of the muscle [20]. In contrast, the relationship between muscle morphology and craniofacial structure has been investigated most extensively in the masseter muscle. A recent systematic review and meta-analysis demonstrated significant associations between masseter thickness, cross-sectional area, and volume and vertical skeletal pattern, with lower values reported in hyperdivergent individuals; however, the available evidence for sagittal classifications was insufficient to permit meta-analysis [6]. Findings from individual studies addressing the sagittal dimension are also inconsistent. A recent study reported greater masseter thickness in Class III individuals than in Class I and II groups [24], whereas another study found the lowest masseter thickness in the Class III group [25]. In addition, a large cohort study including 670 individuals found no significant association between masseter thickness and sagittal malocclusion, while significant relationships were observed with individual factors such as sex and body mass index [26]. Although the mentalis and masseter muscles differ in their anatomical and functional roles, these findings suggest that muscle morphology cannot be explained solely by sagittal skeletal classification. The absence of significant differences in mentalis muscle thickness among the sagittal groups may also be interpreted within this context.

The shear modulus measured at rest primarily reflects the passive mechanical properties and basal tone of the muscle rather than serving as a direct measure of muscle force or activity [27,28]. Within the masticatory system, SWE applications have focused predominantly on the masseter muscle. Higher masseter stiffness has been reported in individuals with temporomandibular disorders [29], whereas resting values in healthy adults have been reported to vary widely, from approximately 10 to 43 kPa [30,31,32]. Nevertheless, the current SWE literature is limited by substantial methodological heterogeneity and low certainty of evidence [33,34]. Beyond elastographic findings, previous studies have highlighted associations between bruxism, temporomandibular symptoms, and psychosocial factors [35,36,37]. However, these clinical and behavioral associations should not be interpreted as direct indicators of muscle stiffness, emphasizing the need to distinguish functional symptoms from objective mechanical measurements.

Among the sagittal skeletal groups, an overall elastographic difference was detected only in the right intermediate region (η^2^ = 0.182). Bonferroni-adjusted pairwise comparisons showed significantly higher values in Class II than in Class I, whereas neither comparison involving Class III reached statistical significance. Moreover, this finding was confined to a single measurement region and was not replicated in the homologous region on the contralateral side. It should therefore be regarded as an exploratory finding rather than evidence of a systematic sagittal skeletal-class effect on mentalis stiffness. The present data cannot determine whether this isolated unilateral finding reflects a true biological difference or technical variability related to factors such as transducer pressure, probe angulation, or measurement positioning [27].

Several FDR-significant associations in the Class III group involved vertical skeletal measures, particularly SN–GoGn and Björk sum. However, these findings should not be interpreted as evidence of a distinct or consistent Class III-specific pattern. SN–GoGn and Björk sum represent related aspects of vertical craniofacial morphology, and elastographic measurements obtained from adjacent regions of the same muscle are likewise not biologically independent. Therefore, the apparent clustering of significant associations may partly reflect correlation among the underlying measurements rather than multiple independent biological relationships. The Spearman sensitivity analyses showed the same direction for the FDR-significant associations, but these findings remain exploratory given the small within-group sample size. A previous study involving patients undergoing orthognathic surgical planning also demonstrated differences in vertical growth patterns across sagittal skeletal groups, highlighting the importance of considering both sagittal and vertical dimensions when interpreting craniofacial relationships [38].

Although mean SN–GoGn and Björk sum values did not differ significantly among the skeletal groups, additional models examining vertical skeletal morphology showed significant group × covariate interactions when either SN–GoGn or Björk sum was included as a covariate. These interactions indicated that the relationship between vertical skeletal morphology and right intermediate elastography varied across sagittal groups and could not be represented by a simple common-slope adjustment. Consequently, the homogeneity-of-regression-slopes assumption was not satisfied, and conventional ANCOVA-adjusted group effects were not interpreted (Supplementary Table S4). These findings further support a cautious interpretation of the relationships between sagittal classification, vertical skeletal morphology, and resting mentalis stiffness.

Although hyperdivergent morphology has generally been associated with reduced muscle thickness and functional capacity in the masticatory muscles [6,8], previous functional studies suggest that the relationship between perioral muscle activity and craniofacial morphology may differ from that observed for the masticatory muscles. Increased activity of the mentalis and other perioral muscles during lip closure has been reported in individuals with lip incompetence, and this has been associated with increased lower anterior facial height and a steeper mandibular plane angle [9,39]. In addition, lower lip closing force has been shown to be greater in Class III individuals with mandibular prognathism than in Class I controls, with positive associations with mandibular plane angle and negative associations with IMPA [40]. These previous findings provide a possible functional context for relationships between vertical craniofacial morphology and the perioral musculature. However, the present resting SWE measurements do not directly assess muscle activity or functional demand. Because lip competence and electromyographic activity were not assessed, no functional mechanism can be inferred from the observed associations.

In the Class I group, limited associations were observed between mentalis stiffness, mandibular incisor position, and upper lip morphology. These findings are consistent with recent evidence suggesting that oral muscle pressure and the balance between perioral and lingual forces may be associated with malocclusion and dentoalveolar relationships [41]. Nevertheless, elastographic stiffness is not equivalent to labial pressure, and because these associations were region-specific, their functional significance should be interpreted cautiously. The associations observed in the Class II group were more heterogeneous and were difficult to explain on the basis of a clear biological mechanism.

Multiple testing and the limited sample size should also be considered when interpreting these correlations. Although the Benjamini–Hochberg procedure reduces the false discovery rate, it does not eliminate the possibility of false-positive findings or unstable associations [42]. Given the sample size of 16 participants per skeletal group, the magnitude of individual correlation coefficients may be sensitive to sampling variability and should therefore be interpreted cautiously. Accordingly, these findings should be regarded as exploratory rather than confirmatory, and differences in significant correlations across skeletal groups should not be interpreted as evidence of distinct group-specific biological mechanisms. The supplementary correlation matrices also showed several nominally significant associations before adjustment, whereas only a subset remained significant after FDR correction. This reduction further supports interpreting the surviving associations as hypothesis-generating rather than confirmatory findings.

Nevertheless, the combined assessment of the morphological and mechanical properties of the mentalis muscle provides a different perspective on the relationship between the perioral musculature and craniofacial structure. The findings suggest that resting mentalis characteristics are not directly related to mandibular symphysis morphology, whereas elastographic properties may show limited associations with vertical skeletal and soft-tissue characteristics. Larger studies incorporating dynamic muscle assessments are needed to clarify the functional significance of these relationships.

This study has several limitations. Its cross-sectional design precludes temporal or causal interpretation of the relationships between the mentalis muscle and craniofacial morphology. The small within-group sample size (*n* = 16 per skeletal group) limits the precision and stability of the correlation estimates and increases their susceptibility to sampling variability. Therefore, despite FDR correction, the observed within-group correlations should be considered exploratory and require confirmation in larger independent samples.

In addition, all ultrasonographic assessments were performed at rest, and participants were not selected or stratified according to lip competence. Formal lip-competence status, quantitative interlabial distance, electromyographic activity, lower-lip closing force, and functional lip behavior during swallowing or speech were not assessed as study variables. Consequently, the present findings cannot directly support or refute a functional role of the mentalis muscle, and the absence of group differences in thickness and most elastography measurements should be interpreted within this limitation. An additional limitation is that sagittal skeletal classification was based on the ANB angle, which may be influenced by cranial-base morphology, vertical skeletal relationships, and mandibular rotation. Although Wits appraisal was recorded as a complementary measure of sagittal jaw relationship, it was not used as an additional criterion for group classification.

Because the groups were defined according to sagittal skeletal pattern, the associations observed with vertical skeletal parameters, particularly in the Class III group, should be confirmed in larger independent samples stratified according to vertical facial pattern. Potential confounding factors such as body mass index, masticatory habits, bruxism, and temporomandibular symptoms were not systematically assessed and therefore could not be accounted for in the analyses. Their potential influence on the elastographic measurements cannot be excluded.

Finally, SWE measurements of a thin superficial muscle such as the mentalis may be particularly sensitive to technical factors, including probe position, angulation, ROI placement, and transducer pressure. Although care was taken to minimize tissue compression during acquisition, probe pressure was not quantitatively standardized. In addition, inter-examiner reliability was not assessed. These technical factors may have contributed to measurement variability and should be considered particularly when interpreting the isolated unilateral elastographic finding.

## 5. Conclusions

The ultrasonographic characteristics of the mentalis muscle showed no consistent association with mandibular symphysis morphology across sagittal skeletal patterns. Mentalis muscle thickness was comparable among the groups, whereas elastographic stiffness demonstrated only a limited and region-specific between-group difference. After correction for multiple testing, selected associations between elastographic stiffness and skeletal or soft-tissue variables remained; however, these exploratory correlations should not be interpreted as evidence of skeletal-class-specific biological mechanisms. Resting SWE reflects mechanical properties rather than direct muscle activity, and the observed associations require confirmation in larger independent samples incorporating functional assessments of the perioral musculature.

## Figures and Tables

**Table 1:** Demographic characteristics of the study groups.

Characteristic	Class I (*n* = 16)	Class II (*n* = 16)	Class III (*n* = 16)	Test Statistic	*p* Value
Age (years) mean ± SD	23.19 ± 3.78	24.81 ± 3.75	24.38 ± 3.44	F = 0.845	0.436
Female, *n* (%)	10 (62.5%)	8 (50.0%)	9 (56.3%)	χ^2^ = 0.508	0.776
Male, *n* (%)	6 (37.5%)	8 (50.0%)	7 (43.8%)		

SD, standard deviation; F, one-way analysis of variance statistic; χ^2^, chi-square statistic.

**Table 2:** Comparison of cephalometric measurements among skeletal groups.

Variable	Class I ^1^ (*n* = 16)	Class II ^2^ (*n* = 16)	Class III ^3^ (*n* = 16)	*p*	η^2^	Post Hoc
	Mean ± SD	Mean ± SD	Mean ± SD			
	95% CI	95% CI	95% CI			
SNA (°)	80.04 ± 2.18	81.20 ± 2.01	80.04 ± 3.38	0.353	0.045	
	78.87–81.20	80.13–82.26	78.23–81.84			
SNB (°)	77.14 ± 2.01	74.65 ± 1.90	81.56 ± 3.69	<0.001	0.551	3 > 1 > 2
	76.07–78.21	73.64–75.66	79.60–83.53			
ANB (°)	2.85 ± 0.84	6.58 ± 1.36	−1.59 ± 1.39	<0.001	0.889	2 > 1 > 3
	2.40–3.30	5.86–7.31	−2.33–−0.85			
Wits appraisal (mm)	0.72 ± 2.27	6.39 ± 2.54	−6.47 ± 3.52	<0.001	0.787	2 > 1 > 3
	−0.49–1.93	5.04–7.74	−8.34–−4.59			
SN–GoGn (°)	33.97 ± 5.62	35.22 ± 6.84	34.97 ± 4.43	0.809	0.009	
	30.98–36.96	31.57–38.86	32.61–37.33			
Björk sum (°)	393.97 ± 5.62	395.22 ± 6.84	394.60 ± 4.48	0.828	0.008	
	390.98–396.96	391.57–398.86	392.22–396.99			
Gonial angle (°)	122.91 ± 6.38	122.45 ± 7.26	128.55 ± 3.36	0.009	0.19	3 > 1, 2
	119.51–126.31	118.58–126.32	126.76–130.33			
IMPA (°)	93.74 ± 5.05	95.91 ± 8.64	82.01 ± 3.64	<0.001	0.513	1, 2 > 3 ^a^
	91.04–96.43	91.31–100.52	80.07–83.95			
Interincisal angle (°)	128.86 ± 7.95	133.93 ± 8.75	134.97 ± 6.32	0.068	0.113	
	124.62–133.09	129.27–138.59	131.60–138.33			
L1-NB (°)	24.87 ± 5.17	25.76 ± 4.59	18.41 ± 2.67	<0.001	0.384	1, 2 > 3 ^a^
	22.11–27.62	23.32–28.21	16.99–19.84			
L1-NB (mm)	5.24 ± 2.10	6.15 ± 1.96	3.06 ± 1.49	<0.001	0.339	1, 2 > 3
	4.12–6.36	5.11–7.20	2.27–3.86			
U1-NA (°)	23.14 ± 3.48	13.97 ± 6.14	27.82 ± 6.50	<0.001	0.535	1, 3 > 2
	21.28–24.99	10.70–17.24	24.36–31.28			
U1-NA (mm)	3.96 ± 1.44	1.72 ± 1.45	5.59 ± 1.90	<0.001	0.508	3 > 1 > 2
	3.19–4.73	0.95–2.49	4.58–6.61			
Overbite (mm)	1.45 ± 2.13	3.17 ± 1.54	0.36 ± 1.93	0.001	0.286	2 > 1, 3
	0.31–2.58	2.35–3.99	−0.66–1.39			
Overjet (mm)	3.61 ± 1.63	4.80 ± 1.44	−1.90 ± 1.29	<0.001	0.809	1, 2 > 3
	2.74–4.48	4.03–5.57	−2.59–−1.21			

SD, standard deviation; CI, confidence interval; η^2^, eta-squared effect size. ^1^ Class I; ^2^ Class II; ^3^ Class III. ^a^ Welch’s ANOVA with Games–Howell post hoc comparison.

**Table 3:** Comparison of soft tissue measurements among skeletal groups.

Variable	Class I ^1^ (*n* = 16)	Class II ^2^ (*n* = 16)	Class III ^3^ (*n* = 16)	*p*	η^2^	Post Hoc
	Mean ± SD	Mean ± SD	Mean ± SD			
	95% CI	95% CI	95% CI			
Lower lip height (mm)	46.01 ± 5.24	45.40 ± 4.26	49.26 ± 6.12	0.094	0.1	
	43.21–48.80	43.14–47.67	46.00–52.52			
Lower lip to E-plane (mm)	−2.96 ± 2.11	−1.29 ± 2.57	−4.08 ± 2.73	0.01	0.185	2 > 3
	−4.08–−1.83	−2.66–0.07	−5.54–−2.63			
Upper lip height (mm)	21.16 ± 4.37	21.63 ± 2.43	20.40 ± 2.49	0.555	0.026	
	18.84–23.49	20.33–22.93	19.07–21.72			
Upper lip to E-plane (mm)	−4.56 ± 1.70	−2.70 ± 2.29	−6.64 ± 1.94	<0.001	0.409	2 > 1 > 3
	−5.47–−3.66	−3.92–−1.48	−7.67–−5.60			
Nasolabial angle (°)	98.55 ± 13.27	105.63 ± 12.54	92.06 ± 14.41	0.023	0.154	2 > 3
	91.48–105.62	98.95–112.31	84.39–99.74			

SD, standard deviation; CI, confidence interval; η^2^, eta-squared effect size. ^1^ Class I; ^2^ Class II; ^3^ Class III.

**Table 4:** Comparison of mandibular symphysis measurements among skeletal groups.

Variable	Class I ^1^ (*n* = 16)	Class II ^2^ (*n* = 16)	Class III ^3^ (*n* = 16)	*p*	η^2^	Post Hoc
	Mean ± SD	Mean ± SD	Mean ± SD			
	95% CI	95% CI	95% CI			
Total symphysis height (Id–Me) (mm)	30.12 ± 3.82	32.06 ± 2.11	30.24 ± 3.77	0.193	0.07	
	28.08–32.16	30.94–33.18	28.23–32.25			
Total symphysis height (Id–Man Pl) (mm)	29.72 ± 3.81	31.38 ± 2.19	28.99 ± 3.58	0.118	0.091	
	27.70–31.75	30.21–32.55	27.08–30.90			
Lower symphysis height (B–Man Pl) (mm)	19.18 ± 2.49	19.58 ± 1.41	18.41 ± 2.76	0.355	0.045	
	17.85–20.51	18.83–20.33	16.94–19.88			
Lower incisor height (Li–Man Pl) (mm)	38.92 ± 4.82	40.21 ± 3.01	37.95 ± 4.45	0.316	0.05	
	36.35–41.48	38.61–41.82	35.58–40.33			
Pg-NB (mm)	2.35 ± 0.59	3.18 ± 1.60	2.31 ± 1.19	0.079	0.107	
	2.03–2.66	2.33–4.04	1.68–2.95			
Pg-Me.B (mm)	4.06 ± 0.69	4.08 ± 0.91	4.23 ± 0.97	0.837	0.008	
	3.69–4.43	3.60–4.57	3.71–4.75			
Upper symphysis width (B–B′) (mm)	7.44 ± 1.49	7.64 ± 1.67	7.28 ± 1.54	0.81	0.009	
	6.64–8.23	6.75–8.53	6.46–8.10			
Lower symphysis width (Pg–Pg′) (mm)	12.99 ± 2.29	14.23 ± 1.81	13.37 ± 2.20	0.245	0.061	
	11.78–14.21	13.27–15.20	12.20–14.53			
Symphysis external angle (Id–B–Pg) (°)	150.02 ± 4.97	145.08 ± 5.23	151.49 ± 6.78	0.007	0.197	3 > 2
	147.37–152.67	142.30–147.87	147.87–155.10			
Symphysis inclination (Id–Me/Man Pl) (°)	81.32 ± 3.51	80.07 ± 6.41	74.10 ± 4.12	<0.001	0.311	1, 2 > 3 ^a^
	79.45–83.19	76.66–83.49	71.90–76.29			

SD, standard deviation; CI, confidence interval; η^2^, eta-squared effect size. ^1^ Class I; ^2^ Class II; ^3^ Class III. ^a^ Welch’s ANOVA with Games–Howell post hoc comparison.

**Table 5:** Comparison of mentalis muscle elastography and thickness measurements among skeletal groups.

Variable	Class I ^1^ (*n* = 16)	Class II ^2^ (*n* = 16)	Class III ^3^ (*n* = 16)	*p*	η^2^	Post Hoc
	Mean ± SD	Mean ± SD	Mean ± SD			
	95% CI	95% CI	95% CI			
Right lateral elastography (kPa)	23.84 ± 8.26	26.46 ± 7.43	25.99 ± 5.59	0.548	0.026	
	19.43–28.24	22.50–30.42	23.01–28.97			
Right intermediate elastography (kPa)	20.94 ± 5.97	26.83 ± 4.85	25.35 ± 5.58	0.011	0.182	2 > 1
	17.75–24.12	24.24–29.41	22.38–28.33			
Right medial elastography (kPa)	22.45 ± 5.21	27.71 ± 6.72	25.41 ± 6.14	0.058	0.119	
	19.68–25.23	24.13–31.29	22.14–28.68			
Left lateral elastography (kPa)	35.08 ± 10.76	32.30 ± 10.04	28.69 ± 6.98	0.167	0.076	
	29.35–40.81	26.95–37.65	24.96–32.41			
Left intermediate elastography (kPa)	30.44 ± 10.73	28.02 ± 5.63	28.42 ± 8.70	0.698	0.016	
	24.72–36.16	25.02–31.02	23.78–33.06			
Left medial elastography (kPa)	25.14 ± 5.67	27.21 ± 7.67	29.74 ± 9.35	0.250	0.060	
	22.12–28.15	23.12–31.30	24.75–34.72			
Right lateral thickness (cm)	0.25 ± 0.08	0.24 ± 0.06	0.26 ± 0.06	0.563	0.026	
	0.20–0.29	0.21–0.27	0.23–0.30			
Right intermediate thickness (cm)	0.26 ± 0.09	0.26 ± 0.06	0.26 ± 0.09	0.984	0.000	
	0.21–0.31	0.23–0.29	0.22–0.31			
Right medial thickness (cm)	0.26 ± 0.09	0.28 ± 0.08	0.26 ± 0.08	0.713	0.015	
	0.21–0.31	0.24–0.32	0.21–0.30			
Left lateral thickness (cm)	0.24 ± 0.10	0.21 ± 0.06	0.23 ± 0.06	0.402	0.040	
	0.19–0.29	0.17–0.24	0.20–0.27			
Left intermediate thickness (cm)	0.22 ± 0.08	0.26 ± 0.08	0.23 ± 0.05	0.315	0.052	
	0.18–0.26	0.22–0.30	0.20–0.26			
Left medial thickness (cm)	0.22 ± 0.06	0.24 ± 0.05	0.20 ± 0.05	0.130	0.087	
	0.19–0.26	0.21–0.27	0.17–0.23			

SD, standard deviation; CI, confidence interval; η^2^, eta-squared effect size; kPa, kilopascal. ^1^ Class I; ^2^ Class II; ^3^ Class III. Post hoc comparisons were performed using Bonferroni adjustment when the overall ANOVA was significant; only significant pairwise comparisons are shown.

**Table 6:** Cephalometric–ultrasonographic correlations remaining significant after FDR correction according to skeletal group.

Group	Variable	Ultrasonographic Variable	r	95% CI	*p*	q	Spearman ρ	Spearman *p*
**Class I**	L1-NB (mm)	Right lateral SWE	−0.828	−0.939 to −0.564	<0.001	0.013	−0.759	<0.001
**Class I**	Upper lip height (mm)	Right lateral SWE	−0.766	−0.914 to −0.436	<0.001	0.049	−0.635	0.008
**Class II**	ANB (°)	Left intermediate SWE	0.846	0.603 to 0.945	<0.001	0.007	0.800	<0.001
**Class II**	SNA (°)	Right intermediate SWE	0.768	0.440 to 0.915	<0.001	0.032	0.759	<0.001
**Class II**	ANB (°)	Left medial SWE	0.766	0.436 to 0.914	<0.001	0.032	0.782	<0.001
**Class III**	SN–GoGn (°)	Right lateral SWE	0.731	0.369 to 0.900	0.001	0.037	0.668	0.005
**Class III**	SN–GoGn (°)	Right intermediate SWE	0.761	0.426 to 0.912	<0.001	0.037	0.709	0.002
**Class III**	SN–GoGn (°)	Left lateral SWE	0.728	0.363 to 0.899	0.001	0.037	0.737	0.001
**Class III**	Björk sum (°)	Right lateral SWE	0.726	0.360 to 0.898	0.001	0.037	0.674	0.004
**Class III**	Björk sum (°)	Left lateral SWE	0.749	0.403 to 0.908	<0.001	0.037	0.731	0.001
**Class III**	Lower lip to E-plane (mm)	Right lateral SWE	−0.737	−0.903 to −0.380	0.001	0.037	−0.718	0.002
**Class III**	Lower lip to E-plane (mm)	Right medial SWE	−0.726	−0.898 to −0.360	0.001	0.037	−0.782	<0.001

r, Pearson correlation coefficient; CI, confidence interval; q, Benjamini–Hochberg false discovery rate-adjusted *p* value; ρ, Spearman rank correlation coefficient. Only correlations remaining significant after FDR correction are presented. Spearman correlations were performed as a sensitivity analysis. The 95% CIs for Pearson correlation coefficients were calculated using Fisher’s r-to-z transformation.

## Data Availability

The data presented in this study are available from the corresponding author upon reasonable request. The data are not publicly available due to privacy and ethical considerations.

## Supplementary Materials

The following supporting information can be downloaded at: https://www.mdpi.com/article/10.3390/diagnostics16213527/s1, Figure S1: Cephalometric landmarks and definitions of the skeletal, dental, and soft tissue measurements used in the study; Table S1: Correlations between cephalometric and ultrasonographic variables in the Class I group; Table S2: Correlations between cephalometric and ultrasonographic variables in the Class II group; Table S3: Correlations between cephalometric and ultrasonographic variables in the Class III group; Table S4: General linear model sensitivity analyses for right intermediate elastography according to vertical skeletal morphology.

## Abbreviations

The following abbreviations are used in this manuscript:

ANOVA	Analysis of variance
EMG	Electromyography
US	Ultrasonography
SWE	Shear wave elastography
