# Supplementary material for: Ultrasonographic and Shear Wave Elastographic Assessment of the Mentalis Muscle: Associations with Mandibular Symphysis and Craniofacial Morphology
Source: Diagnostics (Basel). 2026 Oct 27;16(21):3527. doi: 10.3390/diagnostics16213527 (PMC13761803; doi:10.3390/diagnostics16213527)
Supplement: Supplementary file 1 [file diagnostics-16-03527-s001.zip › diagnostics-4642306-supplementary.pdf]

Supplementary Figure S1. Cephalometric landmarks and definitions of the skeletal, dental, and soft tissue measurements used in the study.

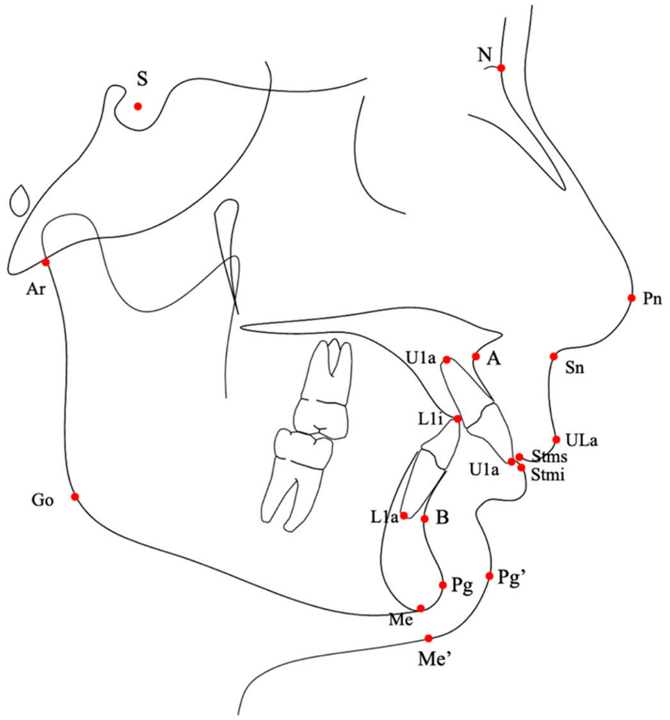

SNA (°): The angle formed between the Sella–Nasion (SN) line and the Nasion–A point (NA) line.

SNB (°): The angle formed between the Sella–Nasion (SN) line and the Nasion–B point (NB) line.

ANB (°): The angle formed between the Nasion–A point (NA) line and the Nasion–B point (NB) line; calculated as the difference between the SNA and SNB angles.

Wits appraisal (mm): The linear distance between points AO and BO, defined as the perpendicular projections of points A and B onto the functional occlusal plane.

Mandibular plane angle (°): The angle formed between the Sella–Nasion (SN) line and the Go–Me mandibular plane.

Björk sum (°): The sum of the saddle angle (N–S–Ar), articular angle (S–Ar–Go), and gonial angle (Ar–Go–Me).

IMPA (°): The angle formed between the long axis of the mandibular incisor and the Go–Me mandibular plane.

L1-NB (°): The angle formed between the long axis of the mandibular incisor and the Nasion–B point (NB) line.

L1-NB (mm): The perpendicular distance from the most labial surface of the mandibular incisor to the Nasion–B point (NB) line.

U1-NA (°): The angle formed between the long axis of the maxillary incisor and the Nasion–A point (NA) line.

U1-NA (mm): The perpendicular distance from the most labial surface of the maxillary incisor to the Nasion–A point (NA) line.

Interincisal angle (°): The angle formed between the long axes of the maxillary and mandibular incisors.

Overjet (mm): The horizontal distance between the incisal edges of the maxillary and mandibular central incisors.

Overbite (mm): The vertical overlap of the maxillary and mandibular central incisors.

Lower lip height (mm): The linear distance between stomion inferius (Stmi) and soft tissue menton (Me′).

Upper lip height (mm): The linear distance between subnasale (Sn) and stomion superius (Stms).

Lower lip to E-plane (mm): The perpendicular distance from labrale inferius (Li) to the esthetic (E) plane.

Upper lip to E-plane (mm): The perpendicular distance from labrale superius (Ls) to the esthetic (E) plane.

Nasolabial angle (°): The angle formed by the columella tangent and the upper lip tangent at subnasale (Sn).

Pg-NB (mm): The perpendicular distance from pogonion (Pg) to the Nasion–B point (NB) line.

Pg-Me.B (mm): The perpendicular distance from pogonion (Pg) to the Me–B line.

Supplementary Table S1. Correlations between cephalometric and ultrasonographic variables in the Class I group

	Right lateral elastography (kPa)	Right intermediate elastography (kPa)	Right medial elastography (kPa)	Left lateral elastography (kPa)	Left intermediate elastography (kPa)	Left medial elastography (kPa)	Right lateral thickness (cm)	Right intermediate thickness (cm)	Right medial thickness (cm)	Left lateral thickness (cm)	Left intermediate thickness (cm)	Left medial thickness (cm)
**SNA (°)**	0.083	-0.124	0.081	0.118	0.389	.662**	-0.089	-0.073	0.13	0.326	0.372	0.365
**SNB (°)**	0.101	-0.159	-0.093	0.022	0.26	.601*	-0.008	-0.063	0.106	0.184	0.218	0.229
**ANB (°)**	0.292	0.286	.524*	.506*	0.373	.578*	-0.256	-0.211	-0.05	0.443	.542*	0.451
**Wits appraisal (mm)**	-0.106	-0.168	0.008	0.319	0.458	0.067	0.211	0.247	0.344	.512*	.575*	.540*
**SN–GoGn (°)**	-0.415	-0.096	-0.262	0.023	0.092	-0.33	0.123	0.105	0.016	-0.263	-0.271	-0.365
**Björk sum (°)**	-0.415	-0.096	-0.263	0.023	0.092	-0.33	0.124	0.106	0.017	-0.263	-0.271	-0.365
**Gonial angle (°)**	-.619*	-0.423	-0.388	-0.161	0.262	-0.197	0.223	0.252	0.234	-0.215	-0.223	-0.278
**IMPA (°)**	-0.22	-0.249	0.075	-0.135	0.089	-0.145	0.207	0.27	0.332	0.216	0.204	0.275
**Interincisal angle (°)**	.692**	0.393	0.132	-0.028	-0.429	0.351	-0.245	-0.373	-0.398	-0.225	-0.223	-0.166
**L1-NB (°)**	-.711**	-0.466	-0.263	-0.204	0.256	-0.368	0.355	0.419	0.434	-0.014	-0.042	-0.044
**L1-NB (mm)**	-.828**	-.546*	-0.479	-0.087	0.362	-0.343	0.367	0.367	0.41	-0.053	-0.017	-0.129
**Overbite (mm)**	0.479	0.276	0.372	0.347	0.165	.543*	-0.217	-0.23	-0.102	0.471	0.48	0.487
**Overjet (mm)**	0.185	0.086	0.069	0.271	0.227	0.222	-0.007	-0.101	0.079	0.341	0.441	0.417
**U1-NA (°)**	-0.322	-0.07	0.027	.528*	.519*	-0.131	0.052	0.126	0.139	0.477	.538*	0.377
**U1-NA (mm)**	-.565*	-0.239	-0.3	0.231	0.353	-0.336	0.355	0.276	0.361	0.264	0.366	0.206
**Lower lip height (mm)**	-0.433	-0.42	-.552*	-0.48	-0.183	-0.248	0.464	0.441	.523*	-0.171	-0.103	-0.1
**Lower lip to E-plane (mm)**	-0.439	-0.44	-0.131	-0.084	0.127	-0.378	0.082	0.148	0.064	-0.007	-0.008	-0.084
**Upper lip height (mm)**	-.766**	-.735**	-.545*	-.584*	0.006	-.510*	.539*	.572*	.670**	-0.179	-0.057	-0.093
**Upper lip to E-plane (mm)**	-0.341	-0.492	-0.039	-0.054	0.239	-0.168	0.047	0.136	0.14	0.144	0.195	0.121
**Nasolabial angle (°)**	-.501*	-.582*	-.676**	-0.101	0.204	-0.016	0.368	0.427	0.452	0.129	0.139	0.097
**Total symphysis height (Id–Me) (mm)**	-.653**	-.594*	-.593*	-0.458	-0.051	-0.392	.580*	.654**	.707**	0.024	0.066	0.062

**Total symphysis height (Id–Man Pl) (mm)**	-.652**	-.586*	-.546*	-0.469	-0.046	-0.376	.553*	.645**	.711**	0.041	0.089	0.08
**Lower symphysis height (B–Man Pl) (mm)**	-0.404	-0.441	-0.213	-.576*	-0.169	-0.117	0.346	.520*	.641**	0.085	0.123	0.159
**Lower incisor height (Li–Man Pl) (mm)**	-.640**	-.537*	-0.486	-.522*	-0.057	-0.348	0.485	.517*	.623**	-0.103	-0.039	-0.048
**Pg-NB (mm)**	0.409	0.493	0.105	0.39	-0.007	0.344	-0.123	-0.209	-0.192	0.11	0.076	0.053
**Pg-Me.B (mm)**	-0.165	-0.312	-0.231	-0.394	-0.243	-0.063	0.391	0.427	.570*	0.017	0.149	0.119
**Upper symphysis width (B–B′) (mm)**	0.189	-0.076	0.294	-0.335	-0.223	-0.006	0.171	0.262	0.328	0.2	0.191	0.338
**Lower symphysis width (Pg–Pg′) (mm)**	-0.149	-0.296	0	-.499*	-0.216	-0.036	0.36	.546*	.626**	0.248	0.202	0.359
**Symphysis external angle (Id–B–Pg) (°)**	0.34	0.249	0	-0.132	-0.333	0.113	-0.035	-0.072	-0.136	-0.178	-0.226	-0.171
**Symphysis inclination (Id–Me/Man Pl) (°)**	-0.079	0.013	.556*	-0.211	0.044	0.143	-0.269	-0.034	0.155	0.221	0.301	0.241

**Values are Pearson correlation coefficients (r). *p < 0.05; **p < 0.01 based on unadjusted p values. Correlations remaining significant after Benjamini–Hochberg FDR correction are presented in Table 6 of the main manuscript.

Supplementary Table S2. Correlations between cephalometric and ultrasonographic variables in the Class II group

	Right lateral elastography (kPa)	Right intermediate elastography (kPa)	Right medial elastography (kPa)	Left lateral elastography (kPa)	Left intermediate elastography (kPa)	Left medial elastography (kPa)	Right lateral thickness (cm)	Right intermediate thickness (cm)	Right medial thickness (cm)	Left lateral thickness (cm)	Left intermediate thickness (cm)	Left medial thickness (cm)
SNA (°)	0.443	.768**	0.208	0.095	0.146	0.282	-0.379	-0.149	-0.405	0.184	-0.176	-0.16
SNB (°)	0.291	.537*	-0.089	-0.228	-0.295	-0.132	-0.168	0.073	-0.24	0.309	-0.017	0.033
ANB (°)	0.497	.610*	.678**	.575*	.846**	.766**	-0.41	-0.334	-0.402	-0.382	-0.464	-.514*
Wits appraisal (mm)	0.046	0.044	0.261	.555*	0.388	0.345	-0.168	-0.221	0.24	-0.216	-0.076	-0.057
SN–GoGn (°)	-0.069	-0.025	-0.251	-0.157	-0.065	-0.035	0.428	0.334	0.005	-0.082	-0.355	-0.191
Björk sum (°)	-0.069	-0.025	-0.251	-0.157	-0.065	-0.035	0.428	0.334	0.005	-0.082	-0.355	-0.191
Gonial angle (°)	0.258	0.213	-0.041	-0.015	-0.088	0.034	.576*	.510*	0.156	-0.192	-0.434	-0.226
IMPA (°)	-0.153	-0.042	0.19	0.012	0.184	0.165	-.662**	-0.488	-0.278	0.194	0.431	0.179
Interincisal angle (°)	.660**	0.366	0.462	0.249	0.238	0.161	.603*	0.322	-0.026	-0.461	-.599*	-0.44
L1-NB (°)	-0.412	-0.011	-0.206	-0.347	-0.007	0.111	-.619*	-0.382	-.525*	0.494	0.391	0.185
L1-NB (mm)	-.507*	-0.161	-0.316	-0.414	-0.146	-0.156	-.576*	-0.475	-0.493	.628**	.513*	0.383
Overbite (mm)	0.272	0.254	0.343	0.211	.506*	0.331	-.565*	-0.446	-0.346	-0.361	-0.244	-0.279
Overjet (mm)	-0.282	-0.163	-0.065	0.123	0.27	0.144	-.620*	-0.378	0.018	-0.051	0.156	0.107
U1-NA (°)	-.515*	-0.435	-0.439	-0.097	-0.333	-0.331	-0.371	-0.105	0.406	0.167	0.446	0.383
U1-NA (mm)	-0.193	-0.18	0.042	0.201	0.176	-0.125	-.531*	-0.359	0.133	0.059	0.277	0.22
Lower lip height (mm)	-0.23	0.033	-0.478	-0.208	-0.413	-0.394	-0.06	0.2	0.227	0.457	0.205	0.315
Lower lip to E-plane (mm)	-.501*	-0.104	-0.495	-0.474	-0.291	-0.248	-.617*	-0.316	-0.379	.534*	0.396	0.251
Upper lip height (mm)	-0.213	0.135	-0.443	-0.293	-0.329	-0.314	-.572*	-0.151	-0.139	0.369	0.2	0.14
Upper lip to E-plane (mm)	-0.262	0.061	-0.352	-0.247	0.01	-0.073	-.608*	-0.163	-0.28	0.09	-0.048	-0.157
Nasolabial angle (°)	-.633**	-0.304	-.700**	-0.336	-.591*	-0.156	0.01	-0.084	0.013	0.379	0.256	0.24
Total symphysis height (Id–Me) (mm)	-0.388	-0.198	-.625**	-0.304	-0.369	-.534*	-0.282	-0.071	0.156	0.357	0.214	0.389

Total symphysis height (Id–Man Pl) (mm)	-0.461	-0.204	-.606*	-0.331	-0.352	-.500*	-0.469	-0.26	-0.002	0.47	0.345	0.433
Lower symphysis height (B–Man Pl) (mm)	0.019	0.091	-0.331	-0.268	-0.324	-0.415	-0.113	0.293	0.137	-0.002	-0.096	-0.032
Lower incisor height (Li–Man Pl) (mm)	-0.471	-0.155	-.562*	-0.35	-0.296	-0.409	-.584*	-0.313	-0.099	.528*	0.403	0.43
Pg-NB (mm)	0.403	0.026	.572*	0.271	0.351	0.08	0.008	0.113	0.224	-0.38	-0.005	-0.005
Pg-Me.B (mm)	0.183	0.052	0.192	0.203	0.383	0.096	-0.341	-0.012	0.105	-.554*	-0.333	-0.309
Upper symphysis width (B–B′) (mm)	-0.294	0.078	-0.314	-0.083	-0.106	-0.016	-.736**	-0.403	-0.191	0.221	0.183	0.057
Lower symphysis width (Pg–Pg′) (mm)	-0.304	0.032	-0.337	-0.06	-0.119	-0.114	-.545*	-0.085	0.013	0.156	0.096	-0.052
Symphysis external angle (Id–B–Pg) (°)	0.172	0.367	-0.153	-0.047	-0.146	0.113	0.314	0.194	-0.18	-0.003	-0.422	-0.303
Symphysis inclination (Id–Me/Man Pl) (°)	-0.447	-0.204	-0.18	-0.139	-0.048	-0.039	-.683**	-.635**	-0.357	0.371	0.467	0.243

**Values are Pearson correlation coefficients (r). *p < 0.05; **p < 0.01 based on unadjusted p values. Correlations remaining significant after Benjamini–Hochberg false discovery rate correction are presented in Table 6 of the main manuscript.

Supplementary Table S3. Correlations between cephalometric and ultrasonographic variables in the Class III group

	Right lateral elastography (kPa)	Right intermediate elastography (kPa)	Right medial elastography (kPa)	Left lateral elastography (kPa)	Left intermediate elastography (kPa)	Left medial elastography (kPa)	Right lateral thickness (cm)	Right intermediate thickness (cm)	Right medial thickness (cm)	Left lateral thickness (cm)	Left intermediate thickness (cm)	Left medial thickness (cm)
SNA (°)	-.573*	-0.376	-0.292	-.498*	-0.283	-0.161	-0.438	-0.405	-0.454	-0.008	-0.446	-0.218
SNB (°)	-.596*	-0.432	-0.256	-0.473	-0.246	-0.299	-0.457	-0.361	-0.457	0.063	-0.321	-0.034
ANB (°)	0.246	0.316	0.012	0.077	0.024	0.333	0.088	-0.117	0.05	-0.231	-0.305	-0.48
Wits appraisal (mm)	-0.213	-0.088	-0.305	-0.475	-0.442	-0.177	0.002	-0.417	-0.01	-.520*	-0.359	0.082
SN–GoGn (°)	.731**	.761**	.603*	.728**	.681**	0.182	0.35	0.393	0.139	0.112	0.134	-0.07
Björk sum (°)	.726**	.657**	.556*	.749**	.646**	0.191	0.325	0.444	0.164	0.102	0.171	-0.106
Gonial angle (°)	0.393	0.133	.545*	.640**	.542*	.555*	0.087	.629**	0.194	0.473	0.451	0.025
IMPA (°)	-0.071	-0.322	-0.389	-0.029	-0.336	0.408	-0.157	-0.21	0.35	-0.294	0.118	0.202
Interincisal angle (°)	0.017	0.232	0.338	0.018	0.174	-0.027	-0.009	0.07	-0.178	0.268	0.074	-0.098
L1-NB (°)	0.235	0.047	0.032	.513*	0.251	.521*	-0.217	0.023	0.179	-0.075	0.064	0.121
L1-NB (mm)	0.254	0.178	0.084	0.47	0.299	0.399	-0.215	-0.074	0.065	-0.1	-0.056	0.085
Overbite (mm)	-.611*	-0.453	-.651**	-.595*	-.550*	-0.437	-0.392	-.741**	-0.227	-0.358	-0.288	0.025
Overjet (mm)	0.363	0.113	0.28	0.455	0.259	-0.116	0.248	0.318	0.376	-0.168	0.414	0.301
U1-NA (°)	-0.119	-0.25	-0.312	-0.209	-0.224	-0.324	0.032	-0.118	0.049	-0.209	-0.082	0.118
U1-NA (mm)	0.092	-0.061	-0.313	-0.165	-0.313	-0.175	-0.103	-0.348	-0.09	-0.311	-0.235	0.001
Lower lip height (mm)	.529*	0.446	0.242	0.34	0.268	-0.05	0.373	0.211	-0.028	0.186	0.12	-0.424
Lower lip to E-plane (mm)	-.737**	-.645**	-.726**	-.554*	-.527*	-0.258	-.517*	-.639**	-0.101	-0.358	-0.288	0.089
Upper lip height (mm)	0.165	0.142	-0.133	0.003	-0.157	0.241	0.157	-0.076	0.192	-0.34	-0.19	-.501*
Upper lip to E-plane (mm)	-.582*	-.635**	-.567*	-0.431	-0.463	0.063	-0.482	-0.365	0.036	-0.275	-0.255	-0.016
Nasolabial angle (°)	0	0.292	0.324	0.269	0.409	0.389	0.045	0.118	-0.007	-0.019	-0.19	-0.222
Total symphysis height (Id–Me) (mm)	0.097	0.138	-0.063	0.021	-0.008	-0.02	0.305	-0.018	0.103	-0.116	-0.004	-0.41

Total symphysis height (Id–Man Pl) (mm)	0.052	0.089	-0.146	-0.03	-0.068	-0.026	0.235	-0.104	0.063	-0.144	-0.058	-0.415
Lower symphysis height (B–Man Pl) (mm)	-0.107	0.015	-0.191	-0.148	-0.13	-0.27	0.16	-0.233	-0.022	-0.179	-0.045	-0.286
Lower incisor height (Li–Man Pl) (mm)	0.11	0.107	-0.192	-0.032	-0.113	-0.063	0.189	-0.145	0.015	-0.148	-0.095	-0.462
Pg-NB (mm)	0.118	0.255	0.186	0.016	0.003	0.01	0.374	0.087	0.485	-0.389	0.162	0.105
Pg-Me.B (mm)	0.141	0.406	0.096	0.01	0.107	-0.38	0.155	-0.285	-0.072	-0.298	-0.172	-0.053
Upper symphysis width (B–B′) (mm)	0.243	0.184	0.016	-0.009	0.003	0.02	0.032	-0.075	-0.268	0.215	-0.153	-.530*
Lower symphysis width (Pg–Pg′) (mm)	0.315	0.367	0.261	0.189	0.191	-0.14	0.277	0.029	-0.139	0.072	0.054	-0.252
Symphysis external angle (Id–B–Pg) (°)	0.072	-0.002	0	-0.015	0.097	-0.288	0.022	-0.009	-0.39	0.339	-0.067	-0.241
Symphysis inclination (Id–Me/Man Pl) (°)	-0.308	-0.322	-.503*	-0.347	-0.389	-0.046	-.498*	-.573*	-0.29	-0.183	-0.371	0.03

**Values are Pearson correlation coefficients (r). *p < 0.05; **p < 0.01 based on unadjusted p values. Correlations remaining significant after Benjamini–Hochberg false discovery rate correction are presented in Table 6 of the main manuscript.

Supplementary Table S4. General linear model sensitivity analyses for right intermediate elastography according to vertical skeletal morphology

Model covariate	Effect	F(df)	p	Partial η²	Overall model F(df)	Model p	R^2	Adjusted R^2	Homogeneity of regression slopes
**SN–GoGn (°)**	Covariate	3.947 (1,42)	0.053	0.086	4.484 (5,42)	0.002	0.348	0.270	Not satisfied
	Group	4.389 (2,42)	0.019	0.173					
	Group × covariate	4.713 (2,42)	0.014	0.183					
**Björk sum (°)**	Covariate	2.610 (1,42)	0.114	0.059	3.714 (5,42)	0.007	0.307	0.224	Not satisfied
	Group	3.329 (2,42)	0.046	0.137					
	Group × covariate	3.359 (2,42)	0.044	0.138					

Separate general linear models were constructed using SN–GoGn and Björk sum as covariates. Each model included skeletal group, the respective covariate, and the group × covariate interaction. Because the group × covariate interaction was statistically significant in both models, the homogeneity of regression slopes assumption was not satisfied. Therefore, conventional ANCOVA-adjusted group effects assuming a common regression slope were not interpreted. Group and covariate effects are presented for transparency within the fitted interaction models. Abbreviations: SN–GoGn, mandibular plane angle relative to the sella–nasion line; Björk sum, sum of the saddle, articular, and gonial angles; df, degrees of freedom; partial η², partial eta-squared; R^2, coefficient of determination; adjusted R^2, adjusted coefficient of determination.
